# Overcoming Challenges: Doxycycline as an Alternative Treatment for Hyponatremia in Managing Syndrome of Inappropriate Secretion of Anti-diuretic Hormone (SIADH) in Small Cell Lung Cancer (SCLC): A Case Report

**DOI:** 10.7759/cureus.42102

**Published:** 2023-07-18

**Authors:** Ragda Imran, Zareen Zia, Ahmed Imran Siddiqi, Waqas Shafiq, Hira Irfan

**Affiliations:** 1 Internal Medicine, Shaukat Khanum Memorial Cancer Hospital and Research Centre, Lahore, PAK; 2 Endocrinology and Diabetes, Shaukat Khanum Memorial Cancer Hospital and Research Centre, Lahore, PAK; 3 Endocrinology, Shaukat Khanum Memorial Cancer Hospital and Research Centre, Lahore, PAK

**Keywords:** tetracycline, doxycycline, small cell lung cancer (sclc), treatment of hyponatremia, syndrome of inappropriate adh secretion

## Abstract

Hyponatremia, a common complication in small cell lung cancer (SCLC), can arise from various causes such as cancer itself, its treatment, paraneoplastic syndrome-induced SIADH secretion (syndrome of inappropriate anti-diuretic hormone secretion), and brain metastasis. While fluid restriction is the initial approach, refractory cases require pharmacological intervention in managing hyponatremia secondary to SIADH. This case report presents doxycycline as an alternative treatment option for a patient with refractory hyponatremia and SCLC with brain metastases, resulting in improved serum sodium levels. However, the use of doxycycline was associated with acute pancreatitis, prompting its discontinuation without establishing a definitive causal relationship. This case report highlights the importance of alternative treatments in resource-limited settings and emphasizes personalized care for hyponatremia in SCLC patients. Doxycycline can be an option, but safety and effectiveness require further study.

## Introduction

Syndrome of inappropriate anti-diuretic hormone (SIADH) secretion, often associated with a paraneoplastic endocrine syndrome with small cell lung cancer (SCLC) is characterized by the excessive release of antidiuretic hormone (ADH), leading to hyponatremia and renal water retention. It can manifest in 15% of patients with SCLC at some point during their illness [1]. The underlying cause is the dysregulated production or activity of ADH resulting in increased reabsorption of water by the kidneys [2].

Diagnosing SIADH involves evaluating the patient's intravascular volume status and laboratory findings. The condition should be considered in individuals with low plasma sodium levels (<134 mmol/L), low plasma osmolality (<275 mOsm/L), elevated urine sodium levels (>20 mmol/L), high urine osmolality relative to plasma osmolality (>100  mOsm/kg), absence of edema or volume depletion, and normal renal, adrenal, thyroid, and pituitary function [3]. 

The treatment of SIADH focuses on managing the underlying cause, such as SCLC, with surgical resection, chemotherapy, or radiation. Hyponatremia resulting from SIADH can be controlled by restricting free water intake or administering hypertonic saline (3%) in severe or symptomatic cases. Additional treatment options include the use of vasopressin receptor antagonists or demeclocycline [3]. 

We present a case of refractory hyponatremia secondary to SCLC with brain metastases. The patient's hyponatremia improved with therapeutic doses of doxycycline, a tetracycline derivative like demeclocycline. 

## Case presentation

A 57-year-old male with a case of SCLC, diagnosed 14 months ago, presented to the emergency department. He had undergone chemotherapy and chemoradiation therapy for the past 8 months and was diagnosed with brain metastasis following an episode of altered mental status and irrelevant behavior five months back. A CT scan of the brain at that time revealed multiple supra and infra-tentorial hyperdense lesions with post-contrast enhancement and surrounding vasogenic edema in the left frontal lobe, right temporo-parietal region, and a small lesion in the right cerebellar hemisphere. The patient received radiation therapy (XRT) to the brain and has been on anti-epileptic medication. 

The patient presented to the emergency department with focal seizures, experiencing two episodes. Laboratory investigations revealed low serum sodium levels (118 mmol/L), low serum osmolality (249 mosmol/kg), and high urine osmolality (491 mosmol/kg) with urinary sodium levels of 136 mmol/L, indicating SIADH. Treatment included restricting free fluid intake to 1 L per day and administering sodium tablets. The patient remained symptom-free, and serum sodium levels improved to 124 mmol/L. The patient was discharged with instructions to continue fluid restriction and scheduled for a follow-up appointment in the endocrinology clinic to monitor their condition. 

Four days after discharge, the patient presented again with headache and vomiting. Physical examination showed a well-oriented middle-aged man with a Glasgow Coma Scale score of 15/15. Neurological examination revealed no abnormalities in cranial nerves, motor system, tone, sensation, or reflexes. The abdomen was soft and non-tender, and clear breath sounds were heard during chest auscultation. Laboratory findings showed hyponatremia (serum sodium: 111 mmol/L), low serum osmolarity (248 mosmol/kg), high urine osmolarity (497 mosmol/kg), and a low spot urine sodium level of 73 mmol/L. 

**Figure 1:**
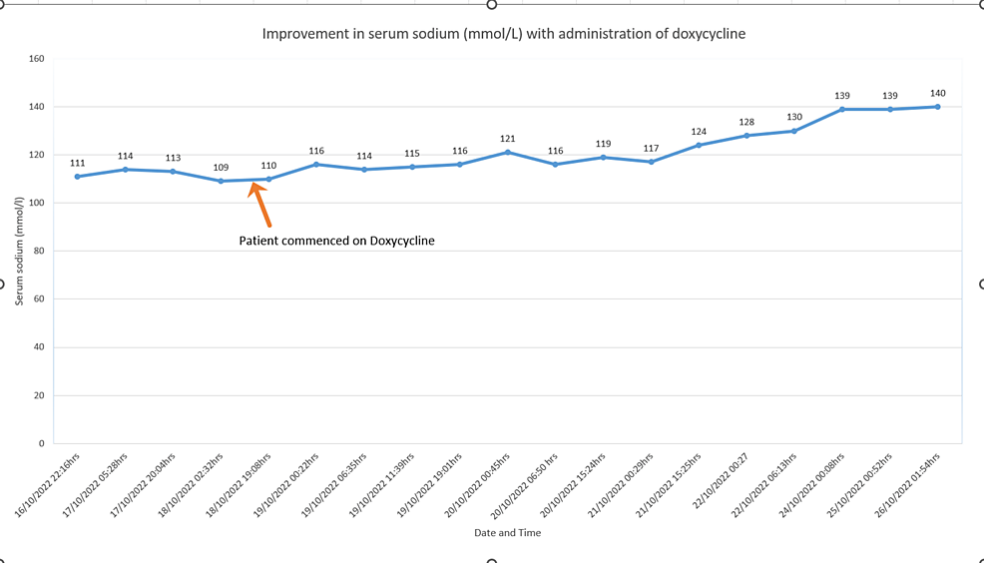
The patient's serum sodium levels improved after starting a trial of doxycycline (100 mg twice daily).

The patient was admitted and first treated with free fluid restriction to 1 L per day and sodium tablets, but his serum sodium levels did not improve. In the absence of definitive therapy options like vasopressin receptor antagonists or demeclocycline, a trial of doxycycline was started, considering its similarity to demeclocycline. The patient received 100 mg of doxycycline twice daily, resulting in a subsequent improvement in serum sodium levels (Figure 1). He was discharged with instructions to monitor daily sodium levels, which remained within the normal range during the follow-up period. 

He visited the endocrinology clinic 1 week after his discharge. His serum sodium levels were in the normal range, and he was advised to continue doxycycline at the same dose. Four days after the endocrinology follow-up, the patient presented to the emergency department with vomiting and upper abdominal pain. His serum sodium was 140 mmol/L, but elevated serum lipase levels of 276.8 u/L raised concerns about pancreatitis associated with doxycycline use. However, no definitive causal relationship was established. Consequently, the decision was made to discontinue the use of doxycycline.

## Discussion

The presented case highlights the challenges in managing hyponatremia associated with SCLC in the absence of the recommended medications. Doxycycline, a tetracycline similar to demeclocycline, was utilized as an alternative due to its potential association with diabetes insipidus [4-5] which can impair urine concentrating ability and help address the hyponatremia. 

The presence of SCLC significantly elevates the risk of hyponatremia, primarily due to factors such as cancer treatment, paraneoplastic syndrome-induced SIADH, and brain metastasis. Accurately distinguishing SIADH from other potential causes is essential to determine the most suitable treatment approach [6]. In the presented case, distinguishing whether the seizures were due to brain metastasis or symptomatic hyponatremia posed a challenge. In the presented case, distinguishing whether the seizures were due to brain metastasis or symptomatic hyponatremia posed a challenge. It is plausible that hyponatremia played a role in lowering the seizure threshold. However, patients' persistently low sodium levels, ranging from 110 to 125 mmol/L over the past three months, indicate a state of chronic hyponatremia. 

In SCLC patients with mild to moderate hyponatremia, initial management often involves fluid restriction [7]. However, if refractory hyponatremia persists pharmacological therapy becomes necessary. In recent trials, vasopressin V2 receptor antagonist tolvaptan is found effective in treating euvolemic and hypervolemic hyponatremia. Vaptans are direct ADH antagonists that produce selective water diuresis (aquaresis) without affecting sodium and potassium excretion. Tolvaptan increases serum sodium levels without the need for water restriction and has a predictable effect with minimal side effects [8]. Vaptans are effective in managing euvolemic hyponatremia related to SIADH, particularly in chronic or minimally symptomatic cases, but they are not recommended for treating asymptomatic hyponatremia [3, 9]. 

Another pharmacological therapy used clinically for the treatment of SIADH is demeclocycline. Demeclocycline exerts its therapeutic effect by inhibiting the responsiveness of ADH in a dose-dependent manner through the blockade of cyclic-AMP generation and action [10]. However, its effectiveness is limited to approximately 60% of patients with SIADH [11]. In vitro and in vivo experiments have shown that tetracycline antibiotics, including demeclocycline, exert their effect by downregulating the ADH-regulated water channel, aquaporin. Among the tetracyclines, demeclocycline exhibits a pronounced effect, while minocycline has a lesser effect, and tetracycline shows only a small impact on the expression of Aquaporin 2 (AQP2) [12]. Interestingly, doxycycline, another member of the tetracycline class, has also been associated with the development of diabetes insipidus (DI) and functional aquaporin deficiency. Case reports have described DI occurring in a patient treated with doxycycline for cellulitis and another patient receiving suppressive antibiotic therapy for left ventricle assist device (LVAD) driveline infections [4-5]. These cases highlight the potential link between doxycycline use and DI, as both patients experienced excessive diuresis following doxycycline administration. 

In the presented case, the patient received 100 mg of doxycycline twice daily, as an alternative treatment when standard treatment options were unavailable. Within 24 h there was a noticeable improvement in serum sodium levels. The patient continued this medication, and later follow-up showed normal serum sodium levels. 

It is important to acknowledge that the use of doxycycline for treating SIADH is an off-label use and should be approached with caution. Individualized dosing and close monitoring of treatment response and adverse effects are important. In this case, the patient was diagnosed with acute pancreatitis, which may have been an incidental finding as no causal relationship was identified. This rare adverse effect has been reported with doxycycline [13]. 

The case emphasizes the usefulness of alternative treatment options in resource-limited settings. However, it is important to weigh the potential benefits and risks of alternative treatments and closely monitor the patient's response to therapy. Collaboration with specialists in managing hyponatremia is crucial to ensure appropriate management and prevent complications. 

## Conclusions

In conclusion, doxycycline can be considered as an alternative treatment option for paraneoplastic SIADH in patients with SCLC when demeclocycline or other specific medications are not suitable. However, cautious dosing and monitoring for adverse effects are necessary. Collaborative decision-making with specialists is recommended for the management of complex cases like this.
